# An Overview of Sub-Cellular Mechanisms Involved in the Action of TTFields

**DOI:** 10.3390/ijerph13111128

**Published:** 2016-11-12

**Authors:** Jack A. Tuszynski, Cornelia Wenger, Douglas E. Friesen, Jordane Preto

**Affiliations:** 1 Department of Oncology, University of Alberta, Edmonton, AB T6G 1Z2, Canada; defriesen@ualberta.ca (D.E.F.); jordane.preto@gmail.com (J.P.); 2 Department of Physics, University of Alberta, Edmonton, AB T6G 2E1, Canada; 3 The Institute of Biophysics and Biomedical Engineering, Faculdade de Ciências, Universidade de Lisboa, Lisboa 1749-016, Portugal; cwenger@fc.ul.pt

**Keywords:** electric fields, biological cells, cancer cells, microtubules, ions, TTFields

## Abstract

Long-standing research on electric and electromagnetic field interactions with biological cells and their subcellular structures has mainly focused on the low- and high-frequency regimes. Biological effects at intermediate frequencies between 100 and 300 kHz have been recently discovered and applied to cancer cells as a therapeutic modality called Tumor Treating Fields (TTFields). TTFields are clinically applied to disrupt cell division, primarily for the treatment of glioblastoma multiforme (GBM). In this review, we provide an assessment of possible physical interactions between 100 kHz range alternating electric fields and biological cells in general and their nano-scale subcellular structures in particular. This is intended to mechanistically elucidate the observed strong disruptive effects in cancer cells. Computational models of isolated cells subject to TTFields predict that for intermediate frequencies the intracellular electric field strength significantly increases and that peak dielectrophoretic forces develop in dividing cells. These findings are in agreement with in vitro observations of TTFields’ disruptive effects on cellular function. We conclude that the most likely candidates to provide a quantitative explanation of these effects are ionic condensation waves around microtubules as well as dielectrophoretic effects on the dipole moments of microtubules. A less likely possibility is the involvement of actin filaments or ion channels.

## 1. Introduction

The effects of external electric fields on biological cells have been extensively studied both in the direct current (DC) and alternating current (AC) cases [1]. In order to elucidate possible impact of electric fields on cells, various experimental assays as well as analytical and computational models have been developed in the past. Experimentally obtained findings were further translated into biomedical applications. While DC or low-frequency AC fields are used to induce stimulation of excitable cells through membrane depolarization or to promote wound healing, high-frequency AC fields are associated with tissue heating and membrane rupture, thus finding its application in diathermy or ablation techniques.

Intermediate-frequency AC electric fields in the kHz to MHz range were commonly assumed to lead to no significant biological effects [1]. However, in a major breakthrough paper, Kirson et al. [2] reported the discovery that low-intensity (1–3 V/cm), intermediate frequency (100–300 kHz) electric fields have a profoundly inhibitory effect on the growth rate of various mammalian tumor cell lines [2,3,4]. This discovery has been translated into a clinical application termed Tumor Treating Fields (TTFields). Based on the results of a Phase III clinical trial [5], TTFields have been approved by the United States Food and Drug Administration (FDA) in 2011 for the treatment of recurrent glioblastoma multiforme (GBM) and their efficacy in treating other solid tumor types is currently being investigated clinically [6]. In late 2015, TTFields were also approved for newly diagnosed GBM patients in combination therapy with temozolomide [7,8] due to significantly increased survival times.

It should be noted that electromagnetic (EM) fields may affect the regulation of cellular growth and differentiation, including the growth of tumors [9,10]. Both static magnetic and electric fields have altered the mitotic index and cell cycle progression of a number of cell types in various species [10]. EM low-frequency fields in the range of 50–75 Hz cause perturbations in the mitotic activity of plant and animal cells and a significant inhibitory effect on mitotic activity occurs early during exposure [11,12,13]. While the field amplitudes used are consistent with those of interest to this report, the frequencies are orders of magnitude lower.

The reduction in the cell number due to an application of TTFields was studied by in vitro experiments with various cancer cell lines. A significant prolongation of mitosis was predicted, where treated cells remain stationary at metaphase for several hours, which was accompanied by abnormal mitotic figures as well as membrane rupture and blebbing leading to apoptosis [2,3]. Furthermore, these experiments showed that the inhibitory effect increases with an increasing electric field intensity, resulting in a complete proliferation arrest of rat glioma cells after 24 h exposure to a field intensity of 2.25 V/cm. Additionally, the effects of TTFields have been shown to be frequency-dependent, with a cancer cell line-specific peak frequency of the maximal inhibitory effect, e.g., 200 kHz for glioma cells [3]. Following these experimental results, two specific mechanisms of action of TTFields have been proposed [2,3,4] which we describe below.

Firstly, the applied field is expected to interfere with proper microtubule (MT) formation preventing a functioning mitotic spindle, due to the force of interactions with the large intrinsic dipole moments of the tubulin dimers [14,15,16] that make up MTs. It has been hypothesized that the tubulin dimers might align parallel to the direction of the applied electric field, rather than along the MT axis. Secondly, the cellular morphology during cytokinesis gives rise to a non-uniform intracellular electric field, with a high density at the cleavage furrow between the dividing cells. This non-uniform field leads to the development of dielectrophoretic (DEP) forces [17] acting on polarizable macromolecules such as MTs, organelles and all charged structures present in the cell, such as ions, proteins or DNA.

Thus, TTFields are considered to be suitable as a novel anti-mitotic cancer treatment modality. In fact, it has been suggested by numerous researchers that endogenous electric fields may play a key role during mitosis. Similar to Cooper [18], Pohl et al. [19] proposed that the onset of mitosis is associated with a ferroelectric phase transition, which establishes an axis of oscillation for the cellular polarization wave. The mitotic spindle apparatus would delineate the polarization field with MTs lined up along the electric field lines. The poles are expected to experience the highest field intensities while the equatorial plane is likely to provide a nodal manifold for the fields. Consequently, the chromosome condensation during this transformation was predicted to be induced by the static dielectric polarization of the chromatin complex as a result of the cellular ferroelectric phase transition. These conclusions have been supported by experimental evidence for peak EM activity during mitosis [20,21] and by physical modeling of the electrostatic forces generated by MTs which generate mechanical force required for chromosome segregation during mitosis and influence chromosomal motion [15,16,22]. A detailed review of this aspect can be found elsewhere [1].

Put together, there is reasonable evidence that especially during mitosis, electric field effects are relevant for the functioning of a dividing cell, especially in the creation of the mitotic spindle. However, to date a rigorous quantitative analysis of the magnitude of these effects within cells exposed to TTFields has not been performed. Furthermore, an analysis of how TTFields might interact with subcellular structures has also never been reported. In a quantitative model, which attempts to explain these effects, an energetic constraint, both from below and above, must be kept in mind. Firstly, for an effect to be of significance at a molecular level, its interaction energy must exceed thermal energy, i.e., kT per degree of freedom (i.e., 4 × 10^−21^ J). Otherwise, thermal fluctuations will disrupt the action of electric fields. Secondly, it must not produce so much thermal energy as to seriously increase the temperature of the cell. In terms of practical comparisons, a cell generates approximately 3 × 10^−12^ W of power (3 × 10^−12^ J/s), much of which is used to maintain a constant physiological temperature. This is found from a simple estimate of energy production by the human body which is 100 W divided by the number of cells in the body which is approximately 3 × 10^13^ [23]. In terms of subcellular forces at work, a minimal amount of useful force at a nanometer scale is 1 pN. Motor proteins generate forces on the order of several pN. A force of 1 pN applied to a tip of a microtubule may be used to bend it by as much as 1 µm [24]. Below, we review electric conduction effects for subcellular structures of interest.

The paper is structured as follows. In Section 2, we review what is known about the shape and intensity of the electric field within cells exposed to externally applied electric fields, focusing on cells during mitosis. As a preparation for following sections, Section 3 offers a general introduction to subcellular electrical conduction and electrostatics. Section 4 and Section 5 are devoted to a comprehensive review of the literature concerned with the effects of electric fields on biopolymers, and with the identification of additional mechanisms by which TTFields might interact with cells. Section 4 covers electric field interactions with the cell membrane and the cytosol, whereas the focus of Section 5 penetrates deeper into the cell, shedding light on the electric field effects on subcellular structures of interest, i.e., microtubules (MTs), actin filaments (AFs), ionic charges and DNA. Finally, in Section 6, we present a discussion about the significance of our findings and about future directions of research that should be undertaken in this area. We hope this paper will set a solid theoretical foundation for future studies into the biophysics of TTFields.

## 2. Induced Electric Fields within Biological Cells in Mitosis

The topic of induced electric fields in and around biological cells subject to DC or AC fields has been investigated for decades. The preliminary and most popular studies on the analytical description of steady-state trans-membrane potential induced on spherical cells go back to the work of H.P. Schwan and colleagues [25]. Arguments were presented to account for the influence of the membrane conductance, surface admittance and spatial charge effects [25], as well as for the geometric and material properties of the cell and the surrounding medium [26]. The impact of external electric fields on a living cell significantly depends on the cell’s shape. Concerning analytical solutions for non-spherical cell shapes, many authors proposed appropriate adaption of the governing equations going back to the work presented in Reference [27]. Later models aimed to study electric polarization effects on oblate and prolate homogeneous and single-shell spheroids have been developed [28]. They were later extended to arbitrarily oriented cells of the general ellipsoidal shape [29]. Importantly, the induced electric field inside a spherical cell is uniform, whereas increasing non-uniformities are predicted for deviations of the regular shape.

Another important aspect is the frequency-dependency of the induced trans-membrane voltage and thus also the intracellular field strength, as predicted by the above-mentioned studies and additional research reported elsewhere [30,31,32,33,34]. For low frequencies, the intracellular space is shielded to a large degree from extracellular electric fields. For example, the electric field strength inside a typical spherical cell is approximately five orders of magnitude lower than that outside the cell [35,36]. However, as the frequency of the field increases, the high membrane field gain diminishes, allowing for higher field intensities to penetrate into the cell.

**Figure 1:**
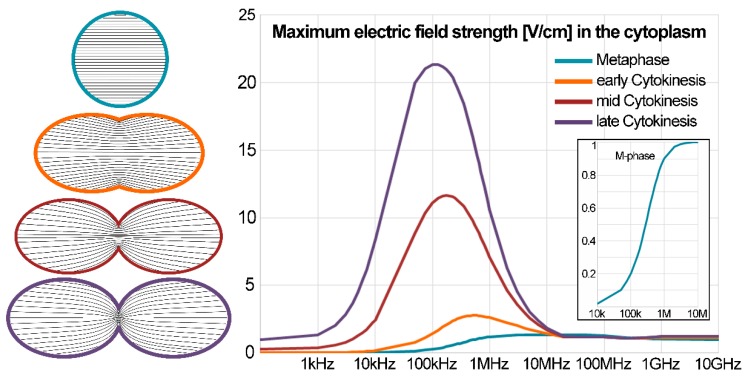
(**Left**) Schematic diagrams of the cell geometries for metaphase and three stages of cytokinesis. Black lines indicate the electric field contours. (**Right**) The maximum intracellular electric field strength in V/cm plotted as a function of field frequency for the four cell shapes subject to an electric field with a 1 V/cm intensity.

Recently, Wenger et al. [37] developed a computational model to study the application of TTFields to isolated cells during mitosis, specifically during metaphase and at different stages of cytokinesis. Comsol Multiphysics (www.comsol.com) was used to solve for the scalar electric potential *V* for frequency ranges between 60 Hz and 10 GHz. With voltages of opposite signs set as boundary conditions, a uniform field of 1 V/cm was induced in the model domain. Following 3D confocal microscopy findings [38,39], the metaphase cell was represented by a sphere with a 10 µm radius and three different stages of cytokinesis were modeled with increased distance between the elliptical mother and daughter cell (see Figure 1, left panel). Three model domains, the extracellular space, the cytoplasm and the membrane, were assigned typical dielectric properties, electrical conductivity and relative permittivity [37].

For a spherical cell during metaphase, the modeling results predict that for frequencies lower than 10 kHz only small changes of the field are detected and the intracellular field strength, *E_i_*, almost equals zero. A first significant increase of *E_i_* is observed at approximately 200 kHz, and *E_i_* increases rapidly as the frequencies increase above this value. This can be seen in the inset of the right panel in Figure 1, which shows a zoomed view of the blue M-phase cell. This transition region depends on the dielectric properties of the cell and its membrane. Nonetheless, above 1 MHz electric current is shunted across the membrane and the impedance is dominated by the cytoplasm. Thus, for an increasing frequency, the electric field inside the cell is augmented and at 1 GHz the cellular structure becomes ‘‘electrically invisible’’ as previously reported [33]. The directions of the electrical field near the cell membrane resemble already predicted results [40].

The model further showed that within the dividing cell the intracellular electric field distribution is non-uniform with highest field intensities at the cleavage plane (black lines in the left panel of Figure 1). These maximum intensities are much higher than the applied field and appear for frequencies in the range 100–500 kHz depending on the stage of cytokinesis, i.e., how far the cell division has already progressed. The corresponding curves are plotted in the right panel of Figure 1, where the highest maximum intracellular field strength of ~22 V/cm is observed for the cell in late cytokinesis.

Due to the inhomogeneity in the electric field distribution, significant dielectrophoretic (DEP) forces are expected to develop within the cell and these DEP forces are thought to be important factors in the mechanism of action of TTFields [41]. This DEP force causes the motion of polarizable particles as a result of the interaction of a non-uniform electric field with their induced dipole moment F=p·∇E [42]. The DEP force is proportional to the volume of the particle *v*, its effective polarizability α and the square of the gradient of the electric field according to: $〈F_{\;DEP}〉=1/2\cdot v\cdot Re\left[\alpha \left(\tilde{E}\cdot \nabla \right){\tilde{E}}^{*}\right]$ using complex phasor notation [42,43]. Thus, the magnitude of the DEP force component is proportional to the magnitude of the gradient of the squared electric field, $\left|F\right|\propto \left|\nabla {\left|E\right|}^{2}\right|\;$in (V^2^/m^3^). The DEP force component showed well-defined peak frequencies at 500, 200, and 100 kHz, respectively, for the three stages considered, from the earliest to the latest stage [37]. This coincides with the peaks of the maximum electric field inside the cell, which are presented in the right panel of Figure 1.

Apart form testing different field intesities, the computational study tested another aspect of TTFields. Namely, it has been shown that the optimal frequency for the inhibitory effect of TTFields is inversely related to cell size [2,44] and that cell volume is increased in almost all cell lines treated with TTFields [45]. The simulation results predicted that the above-mentioned peak frequencies decrease and converge as a function of an increasing cell radius. The corresponding maximum values of the DEP force component also decrease with an increasing cell size with equal decay rates for all cytokinesis stages [37].

In summary, these results obtained by computational modeling confirmed several predicted outcomes of the application of TTFields to biological cells. During metaphase a uniform non-zero *E_i_* is induced. Depending on cell properties, the frequency window of the predicted transition range might be shifted. During cytokinesis, a non-uniform *E_i_* is induced with a substantially increased strength at the cleavage furrow. Frequency-, cell size-, and field-intensity dependences were confirmed.

Experimental validation of the predicted induced field strength values would be of great interest. Electric field strengths have typically only been able to be measured inside membranes with voltage dye and patch-clamp techniques. A promising technique by Tyner et al. [46] reported the generation of a nanovoltmeter that can report local electric fields in the cell and its use would be ideal to calibrate the strength and local distribution of electric fields in the presence of externally applied AC electric fields.

## 3. Subcellular Electrical Conduction and Electrostatics

### 3.1. Protein Conduction

Biological polymers are made up of various proteins, such as actin and tubulin, or nucleic acids as is the case of DNA and RNA. These structures have uncompensated electrical charges when immersed in water but ionic solutions such as the cytoplasm provide a bath of counter-ions that at least partially neutralize the net electric charge. This, however, results in dipolar and higher-moment electric field distributions complicating the situation greatly. Biological water is also believed to create structures with ordered dipole moments and complex dynamics at multiple scales [47], which adds to the complexity of subcellular electric field effects. Additionally, free ions endow the cell with conducting properties along well-defined polymeric pathways as well as in a diffusive way. Membranes support strong electric fields (on the order of 10^5^ V/cm), which, due to counter-ion attraction to charged surfaces in solution, result in Debye screening. This causes an exponential decay of these electric fields on a nm scale [48] but not their complete disappearance when measured in the cell interior (hence a field strength of 10^5^ V/cm decreases to approximately 0.01 V/cm over 100 nm).

The idea that proteins in organisms may have semiconducting properties dates back many decades [49,50] but protein conductivity has been found to be strongly dependent on the hydration state of proteins [51]. Electrical properties of cells and their components were promoted by Szent-Györgyi [52,53], but significant experimental challenges of measuring electric fields and currents at a sub-cellular level impeded progress in this field. The development of more precise experimental tools in the area of nanotechnology holds great promise for rapid progress in the near future [54,55]. Owing to the fact that there have been many previous reviews of electromagnetic effects in biology [1,56,57,58], here we mainly focus on the electrical properties of MTs, actin AFs, ion channels, cytoplasmic ions and DNA with special interest into dynamical electrical properties involving AC fields in the range of 100 kHz. A crucial role of water in the transmission of electrical pulses due to the structure imparted by hydrophilic surfaces [59] is also worth noting. Charge carriers related to protein semi-conduction have largely been electrons, protons as well as ions surrounding proteins in physiological solution. AFs and MTs have been implicated in facilitating numerous electrical processes involving ionic and electronic conduction [60,61] and have been theorized to support dipolar and/or ionic kink-like soliton waves traveling at speeds in the 2–100 m/s range [14,62]. Due to strong coupling between electrical and mechanical degrees of freedom in proteins, mechano-electric vibrations of MTs have been modeled both analytically and computationally [63,64]. Electric fields generated by MTs have been modeled extensively and reviewed recently [65,66,67], although experimental measurement of these fields remains extremely difficult, especially in a live cellular environment.

### 3.2. Electrostatic Interactions Involving Charges and Dipoles of Tubulin

The net charge on a tubulin dimer depends on pH and changes from +5 at pH 4.5 to 0 at pH 5 and drops to −30 at pH 8 [68]. However, in the cytoplasm, a vast majority of electrostatic charges are screened over the distances greater than the Debye length (which varies between 0.6 and 1.5 nm depending on the ionic strength). Therefore, calculating the force due to an electric field of a static electric field with a strength of 1 V/cm acting on a 10 µm-long microtubule, we find from ***F*** = ***qE***, with ***q*** = 10^−13^ C for unscreened charges, that results in ***F*** = 10 pN assuming the field is largely undiminished when penetrating a cell, which is in general a major oversimplification. This latter issue will be addressed at the end of this review. Even if the force is essentially unchanged, the Debye screening of electrostatic charges means that less than 5% of the charge remains exposed to the field resulting in a net force of at most 0.5 pN, most likely insufficient to exert a major influence on the cytoskeleton. If the field oscillates rapidly, the net force would cancel out over the period of these oscillations, i.e., on a time scale of microseconds or less.

The next aspect of MT electrostatics is the effect on the dipole moments of tubulin dimers and of entire MTs. The dipole moment of tubulin (excluding the very flexible and dynamic C-termini which we discuss separately below) has been estimated to be between 566 debye for the α-monomer and 1714 for the β-monomer [69]. However, this is also strongly tubulin-isotype dependent, so these numbers vary a lot between various tubulin isotypes from 500 to 4000 debye [70]. Note that 1 debye is a unit of electric polarization and is equal to 3.33 × 10^−30^ Cm. Therefore, taking the dipole moment of a free tubulin dimer as *p* = 3000 debye as a representative number, we find the interaction energy ***U*** with an electric field of ***E*** = 1 V/cm, and obtain ***U*** *= −p**E***, and hence ***U*** = 10^−24^ J. This is clearly too small (4000 times smaller than thermal energy *kT*) to affect the dynamics of an individual tubulin dimer. However, a single MT contains 1625 dimers per 1 µm of its length, so it could eventually accumulate enough net dipole strength to be significantly affected by the field. Unfortunately, this is very unlikely because of the almost perfect radial symmetry of tubulin dipole arrangements in an MT, which has been predicted by a computer simulation [70]. The individual dipole moments of constituent dimers will almost perfectly cancel out in the radial arrangement of an MT cylinder. There is a small non-cancellation effect along the MT axis but this amounts to less than 10% of the next dipole moment, hence it is doubtful that an entire MT can be aligned in electric fields with intensities lower than 10 V/cm. Unless one uses time-dependent fields (e.g., those used in Reference [71]), much stronger fields are needed for static effects. To put it another way, the torque **τ** between a dipole moment of an MT, ***p***, and an external electric field, ***E***, is proportional to their vector product: **τ** = ***pxE***. For the force to have a meaningful effect on a microtubule, it should exceed 1 pN for lever arm on the order of 1 µm giving a torque of 10^−18^ Nm. With fields on the order of 1 V/cm, and a dipole moment of 3000 debye per tubulin dimer, even if these dipoles were perfectly aligned, it would result in a 1 µm MT only experiencing a torque of 10^−21^ Nm, which is approximately 1000 times too low to be of relevance.

Various special situations involving electrostatic effects on MTs were calculated earlier [68]. Note that a force between a charge and an electric field is given by ***F*** = *q**E***(***x***) where ***E***(***x***) is screened exponentially over the Debye length, which is approximately 1 nm. Hence, a test charge of +5e a distance of 5 nm from the MT surface for a 10-µm MT, experiences a force of 12 pN in water and 1 pN in ionic solution. A tubulin dimer with a dipole of 3000 debye in the vicinity of a microtubule experiences an electrostatic energy of 3 meV. MT-MT interactions due to their net charges with Debye screening accounted for lead to a net force of 9 pN when separated by 40 nm resulting in net repulsion between them. However, at longer distances attractive forces prevail and the corresponding dipole-dipole attraction at 90 nm is only 0.08 pN. The authors of the references [15,16,22] estimated the maximum electrostatic force in the mitotic plate, which was given as ***F*** = 6*n*^2^ pN per MT where *n* is the number of elementary charges on each protofilament. Since ***F*** is estimated to be 1–74 pN for a typical MT, the estimate is 0.4–3.5 uncompensated elementary charges per protofilament. The range of values of the forces involved is certainly within the realm of possible force requirements for chromosome segregation (about 700 pN per chromosome).

### 3.3. MT Conductivity

The building block of an MT is a tubulin dimer, containing approximately 900 amino acid residues with a combined mass of 110 kDa (1 Da is the atomic unit of mass, 1 Da = 1.7 × 10^−27^ kg). Each tubulin dimer in an MT has a length of 8 nm, along the MT cylinder axis, a width of about 6.5 nm and the radial dimension of 4.6 nm. The inner core of the cylinder, known as the lumen, is approximately 15 nm in diameter. MTs have been predicted to exhibit intrinsic electronic conductivity as well as ionic conductivity along their length [72]. MTs have a highly electro-negatively charged outer surface as well as C-terminal tails (TTs), resulting in a cloud of counter-ions surrounding them. Experiment and theory demonstrate that ionic waves are amplified along MTs [72,73]. Since MTs form a cylinder with a hollow inner volume (lumen), MTs have also been theorized to have special conducting properties involving the lumen [55] but there has been no direct experimental determination of the electric properties of the MT lumen. Many diverse experiments were performed to date in order to measure the various conductivities of MTs, with a range of results largely dependent on the experimental method, and this has been reviewed elsewhere [74].

Interestingly, Sahu et al. [75,76] measured conductivity along the periphery of MTs, where the DC intrinsic conductivities of MTs, from a 200 nm gap, were found to be approximately 10^−1^ to 10^2^ S/m. Unexpectedly, MTs at certain specific AC frequencies (in several frequency ranges) were found to be approximately 1000 times more conductive, exhibiting astonishing values for the MT conductivities in the range of 10^3^ to 10^5^ S/m [55,76]. Some resonance peaks for solubilized tubulin dimers were reported as: 37, 46, 91, 137, 176, 281, and 430 MHz; 9, 19, 78, 160, and 224 GHz; and 28, 88, 127, and 340 THz. However, for MTs, the corresponding resonance peaks were given as: 120, 240, and 320 kHz; 12, 20, 22, 30, 101, 113, 185, and 204 MHz; and 3, 7, 13, and 18 GHz. Therefore, for MTs there is some overlap with the 100 kHz range indicating a possible independent confirmation of the sensitivity of MT AC conductivity to this electric field frequency range. These authors showed experimental evidence that the high conductivity of the MT at specific AC frequencies only occurred when the water channel inside the lumen of the MT remained intact [55].

Electro-orientation experiments involving MTs have shown an increased ionic conductivity (0.150 S/m) compared to the buffer solution free of tubulin by as much as 15-fold [77]. MTs exposed to low frequency AC fields (*f* < 10 kHz) exhibit a flow motion due to ionic convection. However, for frequencies above 10 kHz this convection effect is absent. Electric fields with intensities above 500 V/cm and frequencies in the range of 10 kHz–5 MHz, are able to orient MTs in solution. As a point of interest, this frequency range overlaps with the range used by Kirson et al. [2]. However, the intensities of the electric fields used are substantially higher. For instance, a 900 V/cm field with *f* = 1 MHz was able to align MTs within several seconds [77]. Impedance spectroscopy enabled the measurements of the dielectric constant of tubulin as ε = 8.41 [78]. Uppalapati et al. [79] exposed taxol-stabilized MT’s in solution to an AC field, which exhibited electro-osmotic and electro-thermal flow, in addition to MT dielectrophoresis effects. Interestingly above *f* = 5 MHz, electro-hydrodynamic flows were virtually eliminated, and the conductivity of MTs was estimated at 0.25 S/m.

Priel et al. demonstrated MTs’ ability to amplify ionic charge conductivity, with current transmission increasing by 69% along MTs [60], which was explained by the highly negative surface charge density of MTs that creates a counter-ionic cloud subjected to amplification along the MT axis [60]. From Priel et al.’s conductance data, the approximate ionic conductivity of MTs is found to be an astonishing 367 S/m [74]. Below, in the second part of this review, we quantitatively assess AC electric fields on these ionic conductivity experiments, which are expected to be sensitive to the electric field frequencies in the 100 kHz to 1 MHz range.

The multiple mechanisms of MT conductance provide ample possibility to explain the varied reports on MT conductivity in the literature. Ionic conductivity along the outer edge of the MT, intrinsic conductivity through the MT itself, and possible proton jump conduction and conductivity through the inner MT lumen have all been suggested. It is conceivable that TTFields may affect ionic conductivities along MTs as is argued below.

## 4. Collective Effects in the Membrane and Cytoplasm

### 4.1. Membrane Depolymerization Effects

The electric field across the membrane is on the order of 10^5^ V/cm (0.1 V over 8–10 nm), which is 4–5 orders of magnitude greater than TTFields’ amplitude. Therefore, a direct effect of TTFields on cancer cells’ membrane potential is expected to be very minor.

### 4.2. Ion Channel Conduction Effects

Liu et al. [80] reported activation of a Na^+^ pumping mode with an oscillating electric field with a strength of 20 V/cm, which is comparable to the fields of interest in this review, but at a much higher frequency (1.0 MHz) than those of interest. Moreover, neither K^+^ efflux nor Na^+^ influx was stimulated by the applied field in the frequency range from 1 Hz to 10 MHz. These results indicate that only those transport modes that require ATP splitting under the physiological condition were affected by the applied electric fields, although the field-stimulated K^+^ influx and Na^+^ efflux did not depend on the cellular ATP concentration in the range 5 to 800 µM. Computer simulation of a four-state enzyme electro-conformationally coupled to an alternating electric field [81,82] reproduced the main features of the above results.

Channel densities strongly vary among different neuronal phenotypes reflecting different stabilities of resting potentials and signal reliabilities. In model cell types such as in mammalian medial enthorinal cortex cells, modeled and experimental results match best for an average of 5 × 10^5^ fast conductance Na^+^ and delayed rectifier K^+^ channels per neuron [83]. In unmyelinated squid axons counts can reach up to 10^8^ channels per cell. In model channels such as the bacterial KcsA channel one K^+^ ion crosses the channel per 10–20 ns under physiological conductances of roughly 80–100 pS [84], which is consistent with the frequencies of external electric stimulation mentioned above. This allows for a maximum conduction rate of about 10^8^ ions/s. Estimating the distances between the center of the channel pore and the membrane surface to scale along 5 nm and assuming the simplest watery-hole and continuum electro-diffusion model of channels, this would provide an average speed of 0.5 m/s per ion. Ion transition occurs through a sequence of stable multi-ion configurations through the filter region of the channels, which allows rapid and ion-selective conduction [85]. The motion of ions within the filter was intensively studied applying classical molecular dynamics (MD) methods (for a summary see Reference [86]) and density functional studies (e.g., [87]). MD methods used in these simulations solve Newton’s equations of motion for the trajectory of ions.

Time scales for the processes in ion channels can be estimated by the time for translocations (*t_tr_*) between two filter sites separated by ~0.3 nm, i.e., 5 × 10^−10^ s [88] and 5 × 10^−11^ s [87]. Transition rates (from potential mean force maps and the Kramer transition rate model [89] are consistent with these numbers. Changes between a non-conductive and a conductive state in the KcsA occur at a rate of 7.1 × 10^3^ s^−1^, giving a life-time of the non-conducting state of 0.14 ms (~10^−4^ s) [89]. As the duration of the rather (stable) non-conducting state scales in the range 10^−3^–10^−4^ s and the within filter translocation time is on the order of 10^−11^ s, we can expect about 10^7^ filter state changes during a non-conducting state and about 10^10^ switches per second (10 GHz). Consequently, these time scales are incompatible with those resulting from the effects of 100 kHz electric fields (10 μs).

### 4.3. Electric Field Effects on Cytoplasmic Ions

The cytoplasm provides a medium in which fundamental biophysical processes, e.g., cellular respiration, take place. Most biological cells maintain a neutral pH (7.25–7.35) and their dry matter is composed of at least 50% of protein). The remaining dry material is composed of nucleic acids, trace ions, lipids, and carbohydrates. Most of the trace ions are positively charged. A few metallic ions are found which are required for incorporation into metallo-proteins, e.g., Fe^2+^, typically at nanomolar concentrations. In Table 1, we summarize the composition of the cytoplasm regarding the most abundant and important components.

Based on the above, we can estimate the net force on the total charge in the cytoplasm as ***F*** = q***E***, q = 4 × 10^11^ e and ***E*** = 1 V/cm, so the total force is approximately 6 µN, which is sufficient to cause major perturbations in the cell interior. As discussed above, this is strongly depended on the ability of the electric field to penetrate into the cell’s interior, which is easier in the case of non-spherical cells. The net outcome of these ionic oscillations away and towards attractively interacting protein surfaces inside the cytoplasm can be a concomitant series of oscillations of the structures affected by the ionic clouds as schematically shown below.

The viscosity of cytoplasm is approximately η = 0.002 Pa·s [90], hence we can estimate the friction coefficient for an ion in solution as γ = 6πη*r* where r is the ionic radius (hydration shell radius) and find γ = 2 × 10^−12^ Pa·s·m. In an oscillating electric field of amplitude 1 V/cm and a frequency *f* = 200 kHz, an ion’s position will follow periodic motion given by: *x*(*t*) = 0.1·*A*·sin(2π*ft*), i.e., will execute harmonic motion out of phase with the field, with the same frequency and an amplitude A approximately 10% of the radius. However, these ions are simultaneously subjected to the Brownian motion due to their collisions with the molecules of the solvent.

To estimate the effect of an oscillating external electric field on the diffusion of a single biomolecular particle (protein, DNA, simple ion, etc.), the Langevin equation can be written down and solved. In the Ito interpretation [91], the position $X_{t}$ of such a particle is given as a function of time by [92]:

(1) $$dX_{t}=\;\frac{F\left(X_{t}\right)}{\gamma }dt+\sqrt{\frac{2k_{B}T}{\gamma }}dW_{t}$$

where γ is the friction coefficient of the particle, T=310 Κ is the temperature and $k_{B}$ is the Boltzmann constant. The first term on the RHS of Equation (1) accounts for the influence of deterministic forces $F\left(X_{t}\right)$. Assuming there is no interaction other than the coupling with an external electric field $E\left(X_{t}\right)$, we can write $F\left(X_{t}\right)=qE\left(X_{t}\right)$ where q is the net charge of the particle. At intermediate frequencies, i.e., around 100–200 kHz, the wavelength is around 1000 m, which is obviously much larger than the size of a typical cell. Thus, assuming no important changes due to the dissipation of the field, E can be considered almost constant in a cellular environment: $F\left(X_{t}\right)=qE\left(t\right)$. The second term on the RHS represents the random motion, which is due to the many kicks with the surrounding water molecules. Hence, $dW_{t}\;$is usually given by [91]:

(2) $$dW_{t}\sim dt^{1/2}\;\xi \left(t\right)$$

where ξ(t) is a random number, which follows a normal distribution with a mean equal to 0 and a variance equal to 1. Since the Brownian motion is proportional to $dt^{1/2}$, an estimate of dt is needed to evaluate the influence of the external electric field over the thermal noise. The time step dt can be estimated by the time interval between two series of collisions with water molecules, each series being the sum of enough collisions so that the outcome is approximately Gaussian. In other words, one can assume $dt=dx/v_{H2O}$, where dx is the typical separation between two water molecules, i.e., $dx=\sqrt[{3}]{m_{H2O}/\rho_{H2O}}$ where $m_{H2O}$ is the mass of one water molecule and $\rho_{H2O}$ is the mass density of water. Here,$\;v_{H2O}$ is the velocity of water molecules given by $v_{H2O}=\sqrt{3k_{B}T/m_{H2O}}$. The use of the above parameters leads to a typical time step of $dt\sim 5.0\times 10^{-13}\;s$.

The two terms in the RHS of Equation (1) above can be compared to estimate the effect of an electric field over the thermal noise. In the case of a spherical particle, we can assume γ=6πηr, where the hydrodynamic radius is r=1.8 Å and the viscosity of the cytoplasm is η=0.002 Pa·s [90]. By taking q=1 e (a single ion) and $E=E_{0}\cos2\pi ft$ with $E_{0}=1\;V/\mathrm{cm}$, it turns out that the amplitude of the coupling term associated with the electric field is $qE_{0}/\gamma =2.36\;\times \;10^{-6}$ m/s. On the other hand, the noise coefficient is $\sqrt{2k_{B}T/\gamma }\;{\left(dt\right)}^{-1/2}=50.2\;m/s$ when the estimate obtained above is used: $dt\sim 5.0\times 10^{-13}\;$s, which is much larger than the deterministic term. Even in the case of less frequent Brownian collisions, e.g., $dt\sim 10^{-6}\;$s, the noise coefficient is 0.035 m/s which is still much larger than the coupling with the electric field, meaning that an electric field of amplitude 1 V/cm has an exceedingly small probability to influence the diffusion of a single Brownian particle even if the net charge q is 100–1000 times larger as in the case of a protein.

Alternatively, it can be shown that an oscillating electric field at intermediate frequencies with an amplitude of 1 V/cm has no direct sizable effect on the diffusion of biomolecules by considering an ensemble of molecules instead of a single Brownian particle. Assuming a constant electric field E, the distribution of particles as a function of time is given by [93]:

(3) $$P\left(x,t\right)=\;\frac{1}{\sqrt{2Dt}}\exp\left[-\frac{{\left(x\;–x_{0}-\frac{qEt}{\gamma }\right)}^{2}}{2Dt}\right]$$

Here, $D=k_{B}T/\gamma$ is the diffusion coefficient for one particle. From the above equation, a typical time when the particles start to be drifted away because of the electric field is $t=2\left(k_{B}T\right)\gamma /{\left(qE\right)}^{2}$. For a single ion (q=1 e, r=1.8 Å), t=226.1 s, whereas for a typical globular protein (q~100 e, r~1.0 nm), t=0.13 s, which is much larger than the period of an electric field oscillating at hundreds of kHz.

For the sake of simplicity, we have not discussed here how an electric field could induce conformational changes in biomolecular structures, which would affect their charge distributions and dipolar spectra, which, in turn, could modify their diffusion by inducing new interactions with the surrounding molecules. An estimate of such indirect effects would require careful investigations of the studied system based on realistic MD simulations. In this case, the external electric field can be either computationally modeled by initializing the system with added kinetic energy in the directions of the normal modes or by adding an extra coupling term to the force field [94].

## 5. AC Electric Field Effects on Subcellular Structures

### 5.1. Electric Field Effects on MTs

Several experimental efforts were made aimed at measuring the electric field around MTs. Vassilev et al. [71] observed alignment of MTs in parallel arrays due to the application of electric fields with intensities of 0.025 V/cm and of pulsed shape. In cell division, coherent polarization waves have been implicated as playing the key role in chromosome alignment and their subsequent separation [18,19]. Electric fields in the range of 3 V/cm were applied by Stracke et al. [75] to suspended MTs, which moved at pH 6.8 from the negative electrode to the positive one indicating a negative net charge, and an electrophoretic mobility of about 2.6 × 10^−4^ cm^2^·V^−1^·s^−1^. The work of Uppalapati et al. [79] covers the range of frequencies overlapping with TTFields, although the amplitudes are much larger due to the voltage bias of 40 V across a 20-µm gap giving an electric field of 2 × 10^4^ V/cm as opposed to 1 V/cm). Below 500 kHz, MTs flow toward the centerline of electrodes. The *electro-osmotic force* causes the movement of the fluid in a vortex-like manner. This represents the Coulomb force experienced by the ionic fluid due to the applied voltage. The fluid flow velocity *ν* is proportional to the tangential component of the electric field *E_t_*, surface charge density σ, the solution’s viscosity η and the inverse Debye length κ such that: *ν* = *E_t_* σ*/*κη. At lower frequencies, flow velocity is larger. On the other hand, due to strong heating effects of the AC field, the electro-thermal force causes motion of MTs along the length of the electrodes. Above 500 kHz MTs flow toward the gap between the electrodes due to dielectrophoresis. The DEP force experienced by MTs in a non-uniform electric field is given by:

(4) $$〈F_{DEP}〉=\frac{1}{4}\nu \varepsilon_{m}\left[\frac{\omega^{2}\varepsilon_{m}\left(\varepsilon_{p}-\varepsilon_{m}\right)+\sigma_{m}\left(\sigma_{p}-\sigma_{m}\right)}{\omega^{2}\varepsilon_{m}^{2}+\sigma_{m}^{2}}\right]\nabla {\left|E\right|}^{2}$$

where the symbols with subscript “*m*” refer to the medium and “*p*” to the particle. Hence, this process is largely driven by the difference between the conductivities and permittivities of the MTs and the medium, (σ*_p_ −* σ*_m_*) and (ε*_p_ −* ε*_m_*), respectively. We predict that lowering the pH of the solution to the isoelectric point of MTs around pH 5 should substantially reduce this effect and additionally lowering the frequency will reduce it further due to the dependence of the first term on the square of the frequency. At ~5 MHz, the electro-osmotic and electro-thermal flow balance each other out with the flow of MTs being solely due to dielectrophoresis. It is important to compare the dielectrophoretic force to Brownian motion in order to determine whether or not electric fields are sufficiently strong to overcome random motion, i.e., to find out if the dielectric potential exceeds the thermal energy, i.e.,

(5) $$\pi r^{3}\epsilon_{m}\left[\frac{\left(\varepsilon_{p}-\varepsilon_{m}\right)}{\left(\varepsilon_{p}+2\varepsilon_{m}\right)}\right]E^{2}>kT$$

where ε*_m_* is the dielectric constant of the medium and ε*_p_* is the dielectric constant of the particle. ***E*** is the electric field strength and *r* the radius of the particle. Taking as an example a tubulin dimer in solution and the corresponding values of the dielectric constants, one finds that ***E*** must exceed 0.25 V/cm for the field to be effective in orienting polarizable tubulin dimers. Similarly, for a 10-µm long MT we replace the factor π*r*^3^ with π*r*^2^ *L*, where *r* is the radius of a MT (12.5 nm) and *L* its length, to obtain a condition that ***E*** > 0.01 V/cm. Clearly, the electric field values of 1 V/cm (even if they are screened by a large factor inside the cell) are sufficient to exert electrophoretic effects on tubulin and MTs. The longer the MT, the more pronounced the dielectrophoretic effect is predicted to occur.

Recently, Isozaki et al. [95] used MTs labeled with dsDNA to manipulate the amount of net charge and observe the mobility of these hybrid structures compared to control where MTs where only labeled fluorescently with two different tags. It was found for control MTs that the electrophoretic mobility is approximately: 2 × 10^−8^ m^2^·V^−1^·s^−1^ which is consistent with Stracke et al. [75]. For field strengths of approximately 1 V/cm, one can estimate the average velocity of MT translocations as 2 µm/s. They also stated λ*_D_* = 0.74 nm as the Debye length, η = 8.90 × 10^−4^ kg·m^−1^·s^−1^ and *ε* = 6.93 × 10^−10^ C·V^−1^·m^−1^ as the viscosity and dielectric constant of the buffer, respectively. Importantly, they estimated the effective charges of the TAMRA- and AlexaFluor 488-tagged tubulin dimer as 10 e^−^ and 9.7 e^−^, which obviously is only a fraction (approximately 20%–30%) of the vacuum values but much larger than earlier experimental estimates. Electrophoresis experiments were also performed by van den Heuvel et al. [96], with electric field strengths of 40 V/cm, yielding MT electrophoretic mobility in the range of 2.6 × 10^−8^ m^2^·V^−1^·s^−1^, in line with previous reports. They found the effective charge of a tubulin dimer to be approximately 23 e^−^.

### 5.2. Tubulin’s C-Termini Dynamics and AC Electric Fields

**Figure 2:**
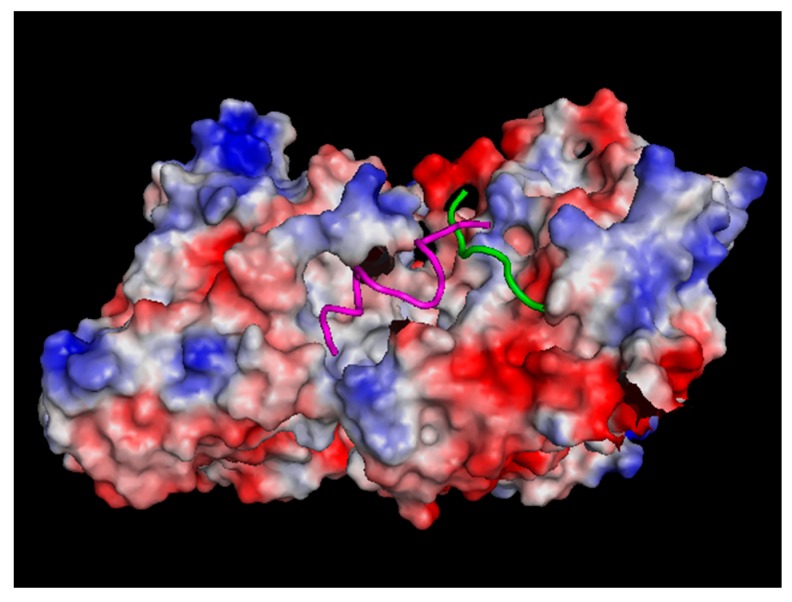
A cross-linked conformation of C-termini stabilizes a straight orientation of a tubulin dimer. A disruption of this conformation can cause MT instability.

Computer simulations demonstrate that ionic waves can trigger C-termini to change from upright to downward conformations initiating propagation of a travelling wave [97]. This wave is predicted to travel as a “kink” solitary wave with a phase velocity of *v_ph_* = 2 nm/ps [97]. A typical time scale for C-termini motion is 100 ps, which is too fast for the 100 kHz frequency range of TTFields. However, C-termini being very flexible and highly charged (with approximately 40% of the tubulin’s charge located there) are likely to dynamically respond to electric fields as local changes of pH are correlated with positive and negative electric field’s polarities, respectively. This effect can cause MT instability as well as interference with motor protein transport as discussed below. A stable dimer conformation is predicted to have C-termini cross-linked between the monomers as shown in Figure 2.

### 5.3. Ionic Waves along MTs and AC Electric Fields

Manning [98] postulated that polyelectrolytes may have condensed ions in their surroundings if a sufficiently high linear charge density is present on the polymer’s surface [99]. The Bjerrum length, λ*_B_*, is defined as the distance at which thermal fluctuations are equally strong as the electrostatic interactions between charges in solution whose dielectric constant is ε at a given temperature *T* in Kelvin. Here, ε_0_ denotes the permittivity of the vacuum and *k_B_* is the Boltzmann constant. Counter-ion condensation occurs when the average distance between charges, *b*, is such that λ*_B_/b* = *S* > 1. In this case, the cylindrical volume of space depleted of ions outside the counter-ion cloud surrounding the polymer functions as an electrical shield. The “cable-like” electro-conducting behavior of such a structure is supported by the polymer itself and the “adsorbed” counter-ions, which are “bound” to the polymer in the form of an ionic cloud (IC). Tuszynski et al. [68] calculated an electrostatic potential around tubulin and extended this to an MT, which demonstrated non-uniformity of the potential along the MT radius with periodically repeating peaks and troughs along the MT axis. Consequently, MTs have been viewed as “conducting cables” composed of 13 parallel currents of ionic flux (corresponding to 13 protofilaments of MTs) and attracting an IC of positive counter-ions close to its surface and along tubulin C-terminal tails (TT), while negative ions of the cytosol are repelled away from the MT surface. The thickness of the negative ion depleted area corresponds to the Bjerrum length. An estimate of the respective condensate thickness λ of the counter-ion sheath for the tubulin dimer (λ*_TD_*) and C-termini (λ*_TT_*) is λ*_TD_* = 2.5 nm and λ*_TT_* = 1.1 nm, as analyzed in [61]. Using a Poisson–Boltzmann approach, the capacitance of an elementary ring of an MT consisting of 13 dimers is found as [100]:

(6) $$C_{0}=\frac{2{\pi \varepsilon }_{0}\varepsilon l}{ln\left(1+\frac{l_{B}}{R_{IC}}\right)}$$

where *l* stands for the length of a polymer unit and *R_IC_* = λ*_TD_* + λ*_TT_* for the outer radius of an IC. For a tubulin dimer: *C_TD_* = 1.4 × 10^−16^ F and for an extended TT: *C_TT_* = 0.26 × 10^−16^ F. Hence:

(7) $$C_{0}=C_{0}+2\times C_{0}=1.92\times 10^{-16}\;F$$

Estimating the electrical resistance for a complete tubulin ring gives *R*_0_ = 6.2 × 10^7^ Ω [60,100]. Including the conductance of both nanopores through an MT surface accounts for the leakage of IC cations into the lumen area and gives a conductance ***G*_0_**, of a ring as ***G*_0_** = **σ_1_** + **σ_2_** = (2.93 + 7.8) nS = 10.7 nS and the corresponding resistivity as ***R*** = 1***/G***_0_ *=* 93 MΩ.

**Figure 3:**
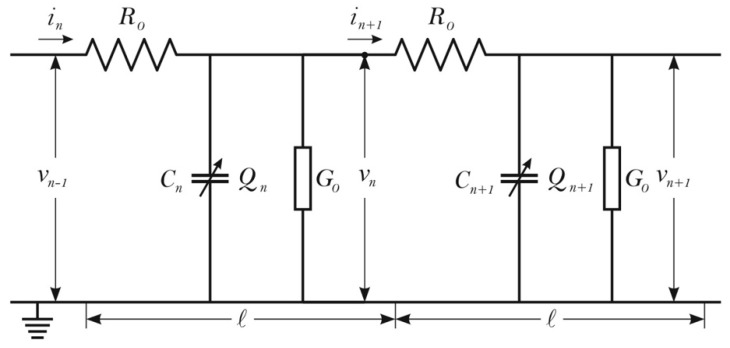
An effective circuit diagram for the n-th unit with characteristic elements for Kirchhoff’s laws applied to a microtubule as an ionic cable [61].

A simple equivalent periodic electric circuit simulating one protofilament of an MT consists of a long ladder network composed of elementary circuit units as shown in Figure 3 [61].

The longitudinal ionic current encounters a series of Ohmic resistors *R*_0_ for each ionic conduction unit (an MT ring). The nonlinear capacity with the charge *Q_n_* for the *n*-th site of the ladder is in parallel with the total conductance *G*_0_ of the two TTs of a dimer. Then using Kirchhoff’s law:

(8) $$i_{n}-i_{n+1}=\frac{\delta Q_{n}}{\delta t}+G_{0}\upsilon_{n},$$

(9) $$\upsilon_{n-1}-\upsilon_{n}=R_{0}i_{n},$$

we find the equations for the voltage propagation:

(10) $$\frac{\delta Q_{n}}{\delta t}=C_{0}\frac{\delta \upsilon_{n}}{\delta t}-C_{0}\Gamma_{0}\Omega \upsilon_{n}-C_{0}\Gamma_{0}\Omega \left(t-t_{0}\right)\frac{\delta \upsilon_{n}}{\delta t}-2b_{0}C_{0}\upsilon_{n}\frac{\delta \upsilon_{n}}{\delta t}$$

Introducing an auxiliary function *u*(*x*, *t*) unifying the voltage and its accompanying IC current as:

(11) $$u_{n}=Z^{1/2}i_{n}=Z^{-1/2}\upsilon_{n}$$

with the characteristic impedance defined as:

(12) $$Z=\frac{1}{\omega C_{0}},$$

leads in the continuum limit to the electric signal propagation equation:

(13) $$-2\frac{\delta u}{\delta x}-\frac{l^{2}}{3}\frac{\delta^{3}u}{\delta x^{3}}-\frac{ZC_{0}}{l}\frac{\delta u}{\delta t}+\frac{ZC_{0}\Gamma_{0}\Omega }{l}\left(t-t_{0}\right)\frac{\delta u}{\delta t}+2\frac{Z^{3/2}b_{0}C_{0}}{l}u\frac{\delta u}{\delta t}-\frac{1}{l}\left(ZG_{0}+Z^{-1}R_{0}-ZC_{0}\Gamma_{0}\Omega \right)u=0$$

The characteristic charging (discharging) time of an elementary unit capacitor *C*_0_ through the resistance *R*_0_ is given by *T*_0_ = *R*_0_*C*_0_ with an estimate for *T*_0_ = 1.2 × 10^−8^ s and the characteristic propagation velocity of the ionic wave: $v=l/T_{0}$ as *v*_0_ = 0.67 m/s*.* A standard travelling-wave with speed *v*, for the normalized function *u*(*x*, *t*)*,* can be used as a solution of the propagation equation, which is a soliton that preserves its width but its amplitude decays over the length of about 400 units corresponding to 3.2 µm, which is of the order of the MT length. Interestingly, a characteristic time for this excitation can readily be estimated as 1.2 × 10^−5^ s whose inverse, the frequency, *f*, is very close to the TTField value, i.e., 90 kHz. The maximum frequency allowed in this model is 68 MHz.

To summarize, ionic conduction along and away from charged protein filaments such as MTs involves cable equations resulting from equivalent RLC circuits surrounding each protein unit in the network. Conduction along the filaments experiences resistance due to viscosity in the ionic fluid. Capacitance is caused by charge separation forming a double layer between the MT surface and ions with a distance separating them comparable to the Bjerrum length. Inductance is caused by helical nature of the MT surface and consequently, solenoidal flows of the ionic fluid along and around the MT. The key numerical estimates of the RLC circuit components are as follows [60]. For a single dimer: *C* = 6.6 × 10^−16^ F, *R*_1_ = 6 × 10^6^ Ω (along the MT), *R*_2_ = 1.2 × 10^6^ Ω (perpendicular to the MT) and *L* = 2 × 10^−12^ H. These numbers can be used to estimate characteristic time scales for the oscillations (LC) and exponential decay (RC) taking place in this equivalent circuit. We obtain for decay times (τ = *RC*) the following values: (a) τ_1_ = 10^−8^ s along the MT length and (b) τ_2_ = 10^−9^ s away from the MT surface. However, due a low value of inductance *L*, the corresponding time for electromagnetic oscillations is found using τ_0_ = (*LC*)^1/2^ as τ_0_ = 0.2 × 10^−12^ s = 0.2 ps. Clearly, the oscillation times are too short for potential effects with 100 kHz-range fields (the time of TTFields oscillations is on the order of 5–10 µs). The decay times are much closer so we will focus on these parameters. Repeating these calculations for a microtubule of length *l*, we note that *R*_1_ scales with length of a microtubule, while *R*_2_ is length independent. The corresponding capacitance in both cases scales with length, therefore τ_1_ scales with length squared (*l*^2^) while τ_2_ scales with length. To obtain actual values, we need to multiply the values for a single ring by the number of rings in an MT. We use the values found for a single ring, i.e., τ_1_ = 10^−8^ s and τ_2_ = 2 × 10^−9^ s and scale them accordingly to estimate the length of MTs that could experience resonant effects in terms of ionic currents along and away from their surface. This way we find the scaling factor that leads to the characteristic times on the order of 10 µs. Therefore, for longitudinal effects, on the order of 50 rings, MTs only 400 nm long would respond to 100 kHz stimulation. On the other hand, for ionic flows pulsating radially around an MT, a 20-µm long MT would be required. These results are very sensitive regarding the choice of parameter values, especially the resistivity where diverse estimates can be found in the literature. In general, there is strong overlap between the time scales of ionic wave propagation and electric field stimulation. It is conceivable that both effects play a role depending on the orientation of the field *vis a vis* the geometry of mitotic spindles and the MTs forming them. It appears that short MTs would be more sensitive to the longitudinal wave generation by TTFields while long MTs should lead to perpendicular wave generation.

Current densities should also be briefly discussed in relation to previously reported endogenous current densities, *j*, in cells, which range from 0.2 to 60 µA/ cm^2^ [101]. This translates into 0.002 < *j* < 0.6 A/m^2^. Since *j* = σ***E*** where ***E*** = 1 V/cm and σ of the cytoplasm has a large range of values reported between 0.1 and 100, we see that even taking the lower limit of 0.1 would result in ionic currents along MTs that would overwhelm the intrinsic ion flows in a dividing cell. It is possible that these externally stimulated currents cause a major disruption of the process of mitosis and associated intra-cellular effects.

It is also worth mentioning that recently metabolic oscillations in cells with a period of approximately 10 to 12 s, were measured in vivo [102] which is many orders of magnitude slower than any AC electric field effects discussed here. Hence, it is safe to assume that there is a very unlikely possibility of electric field effects in the 100 kHz range to interfere with cellular metabolism.

Finally, it is interesting to address the issue of the power dissipated due to a current flowing along an MT. Again, we take as an example a 10 µm-long MT, and we estimate the average power drain as:

(14) $$〈P〉=\left(1/2\right)V_{0}^{2}\left[R/\left(R^{2}+X_{c}^{2}\right)\right],$$

where *X_c_*= 1*/*ω*C* is the capacitive resistance. Substituting the relevant numbers we obtain the power dissipated to be in the 10^−11^ W range which is comparable to the power generated by the cell in metabolic processes (100 W of power generation in the body/3 × 10^13^ cells in the body). Consequently, additional heat generated by these processes may be disruptive to living cells although there is no experimentally detected thermal effect of TTFields.

### 5.4. Resonance Effects on MTs

Cosic et al. [103,104] reported EM resonances in biological molecules (proteins, DNA and RNA) in THz, GHz, MHz and kHz ranges. They proposed the so-called resonant recognition model (RRM) based on the distribution of energy of delocalized proteins in a biological system and charge transfer under resonance with a velocity of 7.87 × 10^5^ m/s and covering distances of 3.8 Å between amino acids, giving a characteristic frequency between 10^13^ and 10^15^ Hz. Then they state a variety of charge transfer velocities yielding different resonant frequencies. Of particular interest to this review is the velocity *v* = 0.0005 m/s which produces EMF in the range of 108–325 kHz for TERT, TERT mRNA and Telomere. This velocity corresponds the propagation of solitons on α-helices. For tubulin and MTs, three specific ranges of resonant frequencies have been predicted by the RRM approach: 97–101 THz, 340–350 THz and 445–470 THz, none of which overlaps with TTField frequencies.

H-bond strength in MTs has been recently computationally estimated [105] as ranging from 11.9 k/mol for the weakest bond to 42.2 kJ/mol for the strongest one and a total of 462 kJ/mol for the α-tubulin/α-tubulin interactions and 472 kJ/mol for the α-tubulin/β-tubulin interactions, which based on the Planck relationship between frequency and energy translates into a range of frequency values between 0.3 × 10^14^ Hz and 1.3 × 10^15^ Hz. Again, these frequencies are much too high to be affected by TTFields. Therefore, we do not expect TTFields to be capable of disrupting the MT structure.

Furthermore, Pizzi et al. [106] measured microwave resonance effects in MTs and found a resonant frequency at 1.510 GHz. This may not correspond to bond-breaking between tubulin dimers but simply to some specific electro-mechanical oscillations. Finally, Preto et al. [92] re-evaluated the Froehlich mechanism for long-range interactions and concluded that classical electromagnetic dipole-dipole interactions at high enough frequencies can lead to attraction between oscillating dipoles over distances comparable to the size of the cell. However, even including a coherently coupled layer of water molecules around a protein, this would require frequencies in the THz range or higher. Consequently, almost all of the resonant frequencies listed above fall well outside the range of potential overlap with the 100 kHz frequencies of TTFields.

### 5.5. Ionic Wave Conductivity along Actin Filaments and AC Fields

AFs are approximately 7 nm in diameter, with a periodic helical structure repeating every 37 nm. Actin filaments are arranged from actin monomers resulting in an alternating distribution of electric dipole moments along the length of each filament [107]. They are characterized by a high electrostatic charge density [108,109] resulting in ionic current conductivity involving the counter-ions surrounding them [109], which is very similar to the effects observed for MTs [60]. The observed wave patterns in electrically-stimulated AFs [30] were very similar to the solitary waveforms recorded for electrically-stimulated non-linear transmission lines [110]. In these experiments [30,42], an input voltage pulse was applied with an amplitude of 200 mV for a duration of 800 ms. Electrical signals were measured at the opposite end of the AF demonstrating that AFs support axial non-linear ionic currents. Since AFs produce a spatially-dependent electric field arranged in peaks and troughs [111] with an average pitch ~35–40 nm, they can be modeled as an electrical circuit with the following non-linear components: (a) a non-linear capacitor associated with the spatial charge distribution between the ions located in the outer and inner areas of the polymer; (b) an inductor; and (c) a resistor, similar to the model described above developed for MTs. A helical distribution of ions winding around the filament at an approximate distance of one Bjerrum length to the filament corresponds to a solenoid in which an ionic current flows due to the voltage gradient between the two ends. For an AF with *n* monomers, its effective resistance, inductance, and capacitance are, respectively:

(15) $$R_{eff}={\left(\sum_{i=1}^{n}\frac{1}{R_{2,1}}\right)}^{-1}+\sum_{i=1}^{n}R_{1,i},$$

(16) $$L_{eff}=\sum_{i=1}^{n}L_{i},$$

(17) $$C_{eff}=\sum_{i=1}^{n}C_{0,i},$$

where *R*_1,*i*_ = 6.11 × 10^6^ Ω, and *R*_2,*i*_ = 0.9 × 10^6^ Ω, such that *R*_1,*i*_ = 7*R*_2,*i*_ [112]. Hence, for a 1-µm length of an AF we find that *R_eff_* = 1.2 × 10^9^ Ω, *L_eff_* = 340 × 10^−12^ H and *C_eff_* = 0.02 × 10^−12^ F. The electrical model of an AF is an application of Kirchhoff’s laws to one section of the effective electrical circuit that is coupled to neighboring monomers. In the continuum limit [112] the following equation describes the spatio-temporal behavior of the electric potential propagating along the actin filament:

(18) $$LC_{0}\frac{\partial^{2}V}{\partial t^{2}}=a^{2}\left(\partial_{xx}V\right)+\;R_{2}C_{0}\frac{\partial }{\partial t}\left(a^{2}\left(\partial_{xx}V\right)\right)-\;R_{1}C_{0}\frac{\partial V}{\partial t}+R_{1}C_{0}2bV\frac{\partial V}{\partial t}.$$

Solitary ionic waves have been described as the solutions of the above nonlinear partial differential equation [112] with an estimated velocity of propagation between 1 and 100 m/s [72]. This model has been recently updated with a more plausible estimation of model parameters [100]. Like MTs [96], AFs can be manipulated by external electric fields [113]. In a similar manner to our analysis of the time scales for MTs as ionic conduction cables with RLC components, we estimate similar time scales for actin and AFs. We readily find for a single actin monomer, that the time scale for LC oscillations is very fast, namely τ_0_ = (*LC*)^1/2^ and τ_0_ = 6 × 10^−14^ s. Secondly, the decay time for longitudinal ionic waves is τ_1_ = *R*_1_*C* = 6 × 10^−10^ s while the corresponding time for radial waves is τ_2_ = *R*_2_*C* = 0.9 × 10^−10^ s. All of the above time scales are not compatible with interactions involving electric fields in the 100 kHz range. However, the situation changes drastically for AFs where there is a similar scaling with the length of the filament as described above for MTs. Taking as an example a 1-µm AF, we find τ_0_ = 10^−11^ s, which is still too short but τ_1_ = *R*_1_*C* = 2.4 × 10^−5^ s which is in the correct range of time for interactions with AC electric fields in the 100 kHz range. It should be noted that AFs have been found sensitive to AC fields under experimental conditions [114].

### 5.6. Electric Field Effects on DNA

Anderson and Record [115] described ionic distribution around DNA in great detail. During interphase, DNA contents present in the nucleus are expected to be protected from external fields due to being enclosed in the nearly spherical nuclear membrane [78]. In addition to the screening effects of being shielded both by the cell membrane and the nuclear wall, the irregular geometry of the DNA strands and their short persistence length indicate that while highly charged, DNA is unlikely to participate in ionic conduction effects shown either for AFs or MTs, both of which have very large persistence lengths.

However, at the beginning of mitosis, the nuclear membrane breaks down, thus potentially not shielding the DNA any longer which would allow for the action of electric fields on chromosomes.

### 5.7. Electric Field Effects on Motor Proteins

Kinesin participates in mass transport along MTs and propagates at a maximum speed of 10^−6^ m/s. This value depends on the concentration of ATP and the ionic concentrations in the medium. In the case of MTs, kinesin transports various crucial cargo and for actin filaments, dynein does the same at similar speeds. Hence each step of a motor protein takes place over the period of a few ms, which is much longer than the period of AC field oscillations. However, kinesin binds to MTs through C-termini, which are very sensitive to electric field fluctuations and hence it is possible that kinesin transport would be very strongly disrupted by these rapid oscillations of C-termini. This aspect merits careful experimental verification.

Another potential member of the cytoskeleton that has been found affected by TTFields [2] is the protein called septin, which are GTP-binding like tubulin but form oligomeric hetero-complexes including rings and filaments. There is no information at the present time that could shed light on the mechanism of TTField effects with septin-based structures.

## 6. Discussion

The cytoskeleton and especially, MTs, may participate in numerous interactions with electromagnetic forces due to the complex charge distribution in and around these protein filaments surrounded by poly-ionic solutions. First of all, there are large net charges on tubulin, which are largely but not completely screened by counter-ions. Secondly, some of the charges are localized on C-termini, which are very flexible leading to oscillating charge configurations. Then, there are ions surrounding the protein that can be partially condensed and susceptible to collective oscillations. Moreover, there are large dipole moments on tubulin and microtubules whose geometric organization importantly affects their response to external fields. Finally, there can be induced dipole moments especially in the presence of electric field gradients. Disentangling the relative importance of the various effects under different conditions is not trivial and requires careful examination.

Depending on the orientation of the electric fields with the cell axis and in particular with the MT axis (however, they fan out from centrosomes in mitotic cells, so there will be at different angles to any field), there could in general be three types of ionic waves generated:

1. Longitudinal waves propagating along the MT surface. In this case each protofilament of a microtubule acts like a cable with its inherent resistance *r*, so the resistance of an entire microtubule would be *R* = *r*/13 since all these cables are in parallel to each other.
2. Helical waves propagating around and along each microtubule, there could be three or five such waves propagating simultaneously mimicking the three-start or five-start geometry of a microtubule. The effective resistance of such cables would be the individual resistance divided by the number of cables in parallel.
3. Radial waves propagating perpendicularly to the microtubule surface.

If a field is oriented at an angle to the MT axis, it is expected that all these wave types may be generated simultaneously. Once AC fields generate oscillating ionic flows, these can in turn:

1. Interfere with ion flows in the cleavage area of dividing cells.
2. Interfere with motor protein motion and MAP-MT interactions.
3. May to a lesser degree affect ion channel dynamics.
4. May in general affect the net charge of the cytoplasm.

Finally, Kirson et al. [2] mention intracellular charged and polar entities such as cytoplasmic organelles as being potentially most directly affected by TTFields. This is not specifically addressed in this paper due to size and scope limitations as well as the scarcity of data in this regard. It has been argued [2] that inhomogeneity in field intensity may exert a uni-directional electric force on all intracellular charged and polar entities, pulling them toward the furrow (regardless of field polarity). It was determined that cytoplasmic organelles are electrically polarized by the field within dividing cells. As a consequence, the TTField-generated forces acting on these organelles may reach values up to 60 pN resulting in their movement toward the cleavage furrow. These organelles can move at velocities up to 30 μm/s and, as a result, they could pile up at the cleavage furrow within a few minutes, interfering with cytokinesis, which may lead to cell destruction. This aspect needs detailed experimental investigation.

Some measurable heating effects in the cytoplasm might also be expected. These fields are not expected to affect permanent dipoles of proteins such as tubulin and actin. Although TTField effects have not been specifically assessed for AFs, an earlier paper [114] investigated exposure of cells to AC electric fields in a low frequency range of 1–120 Hz and found significant induced alterations in the AF structure, which were both frequency- and amplitude dependent. An application of 1–10 Hz AC fields caused AF reorganization from continuous, aligned cable structures to discontinuous globular patches. Cells exposed to 20–120 Hz electric fields were not visibly affected. The extent of AF reorganization increased nonlinearly with the electric field strength. The characteristic time for AF reorganization in cells exposed to a 1 Hz, 20 V/cm electric field was approximately 5 min. Importantly, applied AC electric fields were shown to initiate signal transduction cascades, which in turn cause reorganization of cytoskeletal structures. Therefore, in addition to direct effects of TTFields, there may be indirect, down-stream interactions.

## 7. Conclusions

Based on the extensive analysis of the various possible effects AC electric fields can have on living cells, we conclude the following. Electric field gradients, especially in dividing cells, cause substantial DEP forces on tubulin dimers and MTs. The longer the MT, the more pronounced the effect. Additionally, another likely scenario is that ionic current flows along and perpendicular to MT surfaces (as well as actin filaments, but less likely) take place, which can be generated by AC field oscillations in the 100–300 kHz range. The specific frequency selection depends critically on the length of each filament.

Identification of the strength, cause, and function of intracellular electric fields has only recently been experimentally accessible, although speculations in this area have existed for over a decade. These insights may also assist in devising and optimizing ways and means of affecting cells, especially cancer cells, by the application of external electric fields. With the advent of nanoprobe technology, which has shown promise in measuring these fields at a subcellular level, it is very timely to explore the various physical properties of the cytoplasmic environment including the cytoskeleton and the ionic contents of the cytoplasm. This research promises to contribute to our understanding of the cytoplasm in live cells and the role of microtubules and mitochondria in creating dynamic and structural order in healthy functioning cells. It will also be of help to identify biophysical differences in cancer cells that lead to increased metastatic behavior. Such an understanding may lead to optimized therapies and the identification of specific targets to halt metastatic transformation, as well as insights into the mechanism of action of current electromagnetic cancer therapies that are FDA approved and are in development.

## Figures and Tables

**Table 1:** Composition of the cytoplasm.

Ions	Concentration mM	Non-Ionic Constituents	Concentration mg/mL
K^+^	140	protein	200–300
Na^+^	10	actin	2–8
Cl^−^	10	tubulin (electrolyte)	4
Ca^2+^	10^−4^	pH	~7.2
Mg^2+^	0.5	Specific tissues may differ	Specific tissues may differ

Negative protein charge: 1.6 mol/kg; Positive protein charge: 1.01 mol/kg; Net protein charge: 0.6 mol/kg (−); Net cytoplasm charge: 0.3 mol/kg (−); Potassium ion: 0.5 mol/kg (+); Chloride ion: 0.2 mol/kg (−); Net ion charge: 0.3 mol/kg (+).

## Abbreviations

The following abbreviations are used in this manuscript:

DC	direct current
AC	alternating current
TTFields	Tumor Treating Fields
GBM	glioblastoma multiforme
EM	electromagnetic
MT	microtubule
DEP	dielectrophoretic
AF	actin filament
TT	C-terminal tail
MAP	microtubule associated protein
MD	molecular dynamics
